# Physical Activity among U.S. Preschool-Aged Children: Application of Machine Learning Physical Activity Classification to the 2012 National Health and Nutrition Examination Survey National Youth Fitness Survey

**DOI:** 10.3390/children9101433

**Published:** 2022-09-21

**Authors:** Soyang Kwon, Megan K. O’Brien, Sarah B. Welch, Kyle Honegger

**Affiliations:** 1 Department of Pediatrics, Northwestern University, 225 E Chicago Ave. Box 157, Chicago, IL 60611, USA; 2 Shirley Ryan Ability Lab, Department of Physical Medicine and Rehabilitation, Northwestern University, 710 N. Lake Shore Drive, Chicago, IL 60611, USA; 3 Buehler Center for Health Policy and Economics, Northwestern University, 420 E Superior St., Chicago, IL 60611, USA; 4 Ann & Robert H Lurie Children’s Hospital of Chicago, 225 E Chicago Ave. Box 157, Chicago, IL 60611, USA

**Keywords:** ActiGraph accelerometer, machine learning, walking, early childhood

## Abstract

Early childhood is an important development period for establishing healthy physical activity (PA) habits. The objective of this study was to evaluate PA levels in a representative sample of U.S. preschool-aged children. The study sample included 301 participants (149 girls, 3–5 years of age) in the 2012 U.S. National Health and Examination Survey National Youth Fitness Survey. Participants were asked to wear an ActiGraph accelerometer on their wrist for 7 days. A machine learning random forest classification algorithm was applied to accelerometer data to estimate daily time spent in moderate- and vigorous-intensity PA (MVPA; the sum of minutes spent in running, walking, and other moderate- and vigorous-intensity PA) and total PA (the sum of MVPA and light-intensity PA). We estimated that U.S. preschool-aged children engaged in 28 min/day of MVPA and 361 min/day of total PA, on average. MVPA and total PA levels were not significantly different between males and females. This study revealed that U.S. preschool-aged children engage in lower levels of MVPA and higher levels of total PA than the minimum recommended by the World Health Organization.

## 1. Introduction

Early childhood is an important developmental period for establishing healthy physical activity (PA) habits [1]. In early childhood, PA is particularly important to develop gross motor (GM) skills and acquire movement proficiency [2]. Studies showed that moderate- and vigorous-intensity PA (MVPA) was associated with GM competency among preschool-aged children aged 3–5 years [3,4]. However, according to previous studies [5,6,7,8,9], a large proportion of U.S. preschool-aged children do not engage in recommended levels of PA. Further, a review of publications from 1980 to 2007 [8] and some later studies [5,6,7], but not all [10], have reported that preschool-aged females in particular are less active than preschool-aged males. The World Health Organization (WHO) recently established new PA guidelines for children under 5 years of age [11]. For children 3–4 years of age, the PA guidelines recommend at least 180 min/day of PA at any intensity, of which at least 60 min should be MVPA. To evaluate whether PA health behaviors of U.S. preschool-aged children meet these guidelines, it is critical to collect PA data among preschool-aged children at a national level.

The 2011 U.S. National Health and Examination Survey National Youth Fitness Survey (NNYFS) provides an opportunity to evaluate PA and GM development among U.S. preschool-aged children. The NNYFS conducted accelerometer assessments and the Test of GM Development-2 (TGMD-2) in a representative sample of U.S. children including preschool-aged children. Processed accelerometer data from the NNYFS became publicly available in Monitor-Independent Movement Summary (MIMS) units in 2020. From a previous analysis of the MIMS data from the 2011 NNYFS, we found that daily accumulated MIMS were not different between males and females in U.S. preschool-aged children [12]. However, no PA classification algorithms were available for analyzing MIMS data. Thus, we were unable to evaluate how much time preschool-aged children spent in MVPA and total PA (the sum of light-intensity PA [LPA] and MVPA), which limited our ability to use the MIMS data from the NNYFS to inform public health messages and actions. More recently, NNYFS raw accelerometer data have become publicly available, allowing researchers to apply validated PA classification algorithms.

Using the NNYFS data, we also reported that one in three preschool-aged children (33.9%) scored below average on the overall GM level [13]. Of the two GM subsets (locomotion and object control), locomotor competency was shown to be associated with self-reported participation in specific types of PA (i.e., bike riding, scooter riding, trampoline, soccer, and swimming) [13,14]. However, our prior study [12] that used an overall PA measure (MIMS) that encompassed LPA and MVPA failed to show its positive association with locomotor competency. 

This present paper aims to expand on the findings from our prior studies [12,13], by utilizing a machine learning PA classification algorithm to analyze NNYFS raw accelerometer data. Machine learning is a type of artificial intelligence (AI) that provides the ability to automatically learn/find patterns of complex data from a large amount of data. The machine learning algorithm was shown to have higher accuracy than traditional cut-point methods [15]. The primary aim of this study was to report MVPA and total PA levels in a representative sample of U.S. preschool-aged children, using NNYFS raw accelerometer data. The secondary aim was to examine the associations of MVPA and LPA with GM skills among preschool-aged child participants.

## 2. Materials and Methods

### 2.1. Study Sample 

The study sample included U.S. children 3–5 years of age who participated in the 2012 NNYFS. The 2012 NNYFS was a cross-sectional survey that collected data on PA and fitness levels to provide an evaluation of the health and fitness of children 3–15 years of age living in the U.S. The NNYFS used complex staged and stratified sampling methods to select a representative sample of U.S. children 3–5 years of age (n = 368). Accelerometer data for NNYFS participants were collected using wrist-worn accelerometers; thus, 16 participants with missing arms bilaterally were excluded from accelerometer assessment. Ethical approval and consent were obtained from participants’ parents and guardians. Of 352 eligible participants, 301 (149 females) had accelerometer data for at least 1 day and were included in this analysis. Our prior study [12] reported no difference in participant characteristics between those included and excluded, except that the excluded participants tended to be younger.

### 2.2. Accelerometer Assessment 

ActiGraph GT3X+ accelerometers (Pensacola, FL, USA) were used to assess PA at 80 Hz. During a mobile examination center visit, participants were asked to wear an accelerometer wristband (dorsal orientation) on the non-dominant hand for 9 consecutive days (including 7 full days). Participants returned the accelerometer wristband via mail using pre-paid padded envelopes.

Publicly available raw accelerometer data files were downloaded, from which data for participants 3–5 years of age were extracted. The data cleaning process was identical to that described in our prior study [12]. Briefly, we excluded data collected on day 1 (when the accelerometer was placed on the participant) and day 9 (when the accelerometer was removed from the participant, which occurred in the morning). We further excluded data collected between 10 PM and 6 PM (nighttime sleep period), which resulted in a maximum of 16 h of accelerometer data per full day of wear [16]. To be considered as a valid wear day, each day should have ≥600 wear minutes. Detailed information about the wear/non-wear detection algorithm can be found in the NNYFS Analytic Notes [17]. We included any participants who had ≥1 valid wear day for data analysis [12]. 

To process and analyze the wrist accelerometer data, we applied a validated random forest PA classification algorithm developed for preschool-aged children by Trost and the colleagues [15,18]. The algorithm development and validation have been described in prior publications [15,18]. Briefly, recognizing the low performance of laboratory-trained PA classifiers for free-living data [19], random forest PA classifiers were trained using free-living accelerometer data among preschool-aged children. Tri-axial accelerometer signals were transformed into a single-dimension vector magnitude, and time and frequency domain (base) features as well as temporal features were used to train random forest classifiers with various window sizes: 1, 5, 10, and 15 s [18]. Among the wrist random forest classifiers trained, a 15-s window classifier with both base and temporal (base + temporal) features was reported to have the highest performance for predicting five activity classes: “run”, “walk”, “other MVPA”, ‘LPA”, and “sedentary” (weight average F-score = 81%) [18]. Therefore, we selected the 15-s base + temporal feature classifier to analyze NNYFS wrist accelerometer data. 

The NNYFS wrist accelerometer data were segmented into 15-s non-overlapping windows. Feature extraction was performed using the R script obtained from the model developer [18]. Using the R script for the 15-s base + temporal feature classifier (https://github.com/QUTcparg/PS_PAClassification; accessed on 20 September 2022), each of the 15-s windows was predicted as “run”, “walk”, “other MVPA”, ‘LPA”, or “sedentary”. We calculated the number of windows for each activity class a day. The number of windows for each activity class was divided by 4 to express the time spent in each activity in minutes a day. For example, 40 15-s windows predicted as “walk” a day were converted to 10 min of “walk” a day. Multiple days of estimated time spent in each activity were averaged per participant. MVPA (minutes/day) was calculated by summing minutes spent in “run”, “walk”, and “other MVPA”. Total PA (minutes/day) was calculated by summing time spent in MVPA and LPA. Participants who engaged in MVPA ≥ 60 min/day were considered to have met the WHO MVPA recommendation. Similarly, participants with total PA ≥ 180 min/day were considered to have met the WHO total PA recommendation. 

### 2.3. Gross Motor Assessment 

GM skills were evaluated using the TGMD-2 [20]. The TGMD-2 is a widely accepted tool to evaluate GM among young children that includes two GM subtests: locomotor and object control. The detailed TGMD-2 assessment protocol is described in a prior publication [13] as well as on the NYFS website [21]. In accordance with the TGMD-2 manual [20], a locomotor standard score (range: 1 to 20) and an object control standard score (range: 1 to 20) were calculated. 

### 2.4. Statistical Analyses

We used SAS 9.4 (Cary, NC, USA) for data analyses. We incorporated the complex sampling design of the NNYFS in analyses. To achieve the primary aim, we calculated the means and 95% confidence intervals (CIs) of the PA metrics. We repeated this analysis, separately, for participant characteristics, such as biological sex, age (3, 4, or 5 years), race and ethnicity (Hispanic, non-Hispanic White, non-Hispanic Black, or Other), language spoken at home (English only or at least some non-English), family income levels (ratio of family income to poverty, reported as <1.0 [below the poverty level], 1.0 to <3.0, or ≥3.0) [12]. A correlation coefficient (*r*) between MVPA and LPA was calculated. Frequency analyses were conducted to identify the proportion of children meeting the WHO MVPA and total PA recommendations. 

To achieve the secondary aim, we conducted multivariable linear regression analyses. Independent variables of interest included MVPA and LPA. Dependent variables included locomotor and object control standard scores. Covariates were selected based on our prior analysis [13]: sex, age, family income, and living with a child(ren) ≤5 years of age for the locomotor outcome; and sex, age, and living with a child(ren) 6–17 years of age for the object control outcome. 

## 3. Results

Among 301 participants, accelerometer wear time was on average 915 min (standard error: 7 min). Wear time did not differ by sex, age, or racial/ethnic group. As shown in Table 1, U.S. preschool-aged children were estimated to run for 4 min/day (95% CI = 3, 5) and walk for 14 min/day (95% CI = 13, 15) on average. MVPA was estimated at 28 min/day (95% CI = 25, 30) and LPA was estimated at 361 min/day (95% CI = 343, 378). MVPA and total PA tended to be higher in older preschool-aged children. MVPA and total PA levels did not differ by sex, racial and ethnic group (Table 1), language spoken at home, or family income (Supplementary Table S1). The correlation coefficient *r* between MVPA and LPA was 0.53 (*p* < 0.01). Only 2% of participants met the WHO MVPA recommendation, while 95% met the WHO total PA recommendation. 

Table 2 presents the multivariable linear regression analysis results. An additional 10 min spent in MVPA was significantly associated with a 0.7-point higher locomotor standard score (95% CI = 0.4, 1.0). However, LPA was not statistically significantly associated with a locomotor standard score. Similarly, an additional 10 min spent in MVPA was significantly associated with a 0.4-point higher object control standard score (95% CI = 0.04, 0.7). However, LPA was not statistically significantly associated with an object control standard score.

## 4. Discussion

This study utilized a recently validated machine learning algorithm to estimate free-living PA levels among preschool-aged children. Applying the algorithm, this study identified an estimated national MVPA level among preschool-aged children of 28 min/day, which is much lower than the WHO recommendation of 60 min/day, and an estimated total PA level of 361 min/day, which is much higher than the WHO recommendation of 180 min/day. This study also confirmed that MVPA and LPA levels were not different between preschool-aged males and females. MVPA, but not LPA, was associated with better scores on locomotor and object control skills. 

This study was innovative in the application of an accelerometer-based machine learning PA classification algorithm that is more accurate (weighted kappa = 0.72) than available cut-point algorithms (weighted kappa = 0.31–0.44) [15]. Accelerometer activity monitors have vastly facilitated the evolution of child PA research over the past three decades, allowing researchers to objectively assess free-living PA levels. Using limited raw data, researchers have established accelerometer count cut-points to define PA intensities/classes (i.e., sedentary, light, moderate, and vigorous). However, significant limitations of the cut-point approach, including low accuracy and the inability to detect activity type, have also been recognized. To address these limitations, a new analytic approach, machine learning, has been adopted in PA measurement research. Machine learning can process abundant raw tri-axial acceleration signal data for pattern recognition. To date, the machine learning PA classifier by Trost and the colleagues [15,18] is the most rigorously validated accelerometer-based algorithm for preschool-aged children, which was utilized in this study to estimate PA levels among preschool-aged children. 

This is one of the first studies to report key PA indicators among U.S. preschool-aged children at the national level. Our national estimation of 28 min/day MVPA indicates that many U.S. preschool-aged children do not sufficiently engage in MVPA. However, in interpreting the study results, caution should be taken to consider the direction (i.e., over- or under-estimation) and the size of potential misclassification errors. In a study by Ahmadi and Trost [15], the confusion matrix of the PA classifier indicated that the machine learning algorithm correctly classified 87.5% of LPA and misclassified only 4.7% of LPA as sedentary behavior and only 7.8% of LPA as MVPA. However, a large proportion of MVPA (31.4%) was misclassified as LPA [15]. Altogether, the algorithm slightly over-estimated LPA (140 min by prediction vs. 132 min by the ground truth) and slightly under-estimated MVPA (42 min by prediction vs. 47 min by the ground truth). Therefore, we assume that our MVPA estimate may be underestimated, and LPA estimate may be overestimated. 

It is an important finding that MVPA, but not LPA, was positively associated with GM skills. For GM development and other health benefits, MVPA should be encouraged among preschool-aged children. However, it is concerning that many preschool-aged children do not engage in sufficient levels of MVPA. Our evaluation revealed that preschool-aged children on average walked or ran for 18 min a day and engaged in MVPA for 28 min a day. We suggest that US preschool-aged children should be offered greater opportunities to engage in specific PA types that help GM development, such as bike riding, scooter riding, trampoline, soccer, and swimming [13,14]. In addition, although there are no PA guidelines specific to walking and running activities, we suggest that US preschool-aged children should be encouraged to engage in more walking and running activities throughout the day. Spending more time outdoors [12] and increasing opportunities for walking/running, as opposed to being restrained in a stroller or a car [11], would likely increase levels of MVPA to align with WHO recommendations.

The limitation of this study includes that the cross-sectional examination for a relationship between MVPA and GM skills cannot establish a temporal relationship; thus, we are unable to determine whether limited engagement in MVPA hinders GM development or limited GM competency restricts participation in MVPA. Second, PA levels among preschool-aged children may have changed over the past decade since 2012. In particular, child PA may have decreased during the COVID-19 pandemic [22]. Therefore, our PA estimation based in 2012 data may not accurately reflect the current status of PA among U.S. preschool-aged children. Nonetheless, this study used the most recent available accelerometer data to evaluate key PA indicators among U.S. preschool-aged children at a national level.

## 5. Conclusions

Our evaluation of PA among preschool-aged children using the most accurate PA classification approach available showed that on average, U.S. preschool-aged children engaged in 28 min/day of MVPA and 361 min/day of total PA. We also found that time spent in MVPA, but not in LPA, was positively associated with GM skills. 

## Figures and Tables

**Table 1:** Estimated daily minutes spent in physical activity in U.S. preschool-aged children. The 2012 NNYFS.

	Run	Walk	Other MVPA	LPA	MVPA ^a^	Total PA ^b^
	Weighted mean (95% CI), minutes/day					
All (n = 301)	4 (3, 5)	14 (13, 15)	10 (8, 11)	361 (343, 378)	28 (25, 30)	388 (368, 408)
Sex						
Male (n = 152)	5 (4, 6)	14 (12, 16)	10 (9, 12)	361 (334, 387)	29 (25, 33)	389 (359, 419)
Female (n = 149)	3 (2, 4)	15 (13, 16)	9 (7, 10)	360 (342, 379)	27 (24, 29)	387 (367, 407)
Age						
3 years (n = 96)	3 (2, 4)	13 (10, 16)	8 (6, 10)	333 (300, 366)	24 (19, 29)	357 (320, 393)
4 years (n = 101)	4 (3, 5)	14 (12, 16)	10 (6, 12)	370 (346, 394)	28 (25, 31)	398 (372, 424)
5 years (n = 104)	4 (3, 5)	16 (14, 18)	11 (9, 12)	376 (361, 392)	31 (27, 34)	407 (389, 425)
Race/ethnicity						
Non-Hispanic White (n = 121)	4 (2, 5)	13 (12, 14)	9 (8, 11)	362 (336, 388)	26 (23, 29)	388 (359, 416)
Hispanic (n = 102)	4 (3, 5)	16 (13, 20)	10 (8, 12)	364 (340, 387)	31 (26, 35)	394 (368, 421)
Non-Hispanic Black (n = 61)	4 (3, 5)	15 (13, 17)	11 (9, 12)	368 (342, 395)	29 (26, 33)	398 (368, 427)
Other (n = 17)	4 (2, 5)	13 (7, 19)	8 (5, 12)	320 (275, 364)	25 (15, 35)	345 (292, 398)

Physical activity types were classified based on the random forest activity classification algorithm by Ahmed and Trost [15]. ^a^ Sum of run, walk, other MVPA. ^b^ Sum of MVPA and LPA. CI, confidence interval; LPA, light-intensity physical activity; MVPA, moderate- and vigorous-intensity physical activity; PA, physical activity.

**Table 2:** Multivariable linear regression models for locomotor and object control standard scores in U.S. preschool-aged children. The 2012 NNYFS.

Predictors	Locomotor Standard Score	Object Control Standard Score
	Estimate (95% CI)	Estimate (95% CI)
Intercept	7.6 (6.1, 9.1)	8.2 (6.6, 9.9)
Sex: female vs. male	1.3 (0.7, 2.0)	0.1 (−0.6, 0.8)
Age: 4 vs. 3 years	0.8 (0.1, 1.5)	−0.3 (−1.1, 0.4)
Age: 5 vs. 3 years	0.4 (−0.4, 1.2)	−0.01 (−0.9, 0.9)
Ratio of family income to poverty: 1.0 to <3.0 vs. ≥3.0	0.7 (−0.3, 1.7)	NA ^a^
Ratio of family income to poverty: <1.0 vs. ≥3.0	0.3 (−0.6, 1.1)	NA ^a^
Living with child(ren) ≤5 years of age: yes vs. no	0.3 (−1.1, 1.6)	NA ^a^
Living with child(ren) 6–17 years of age: yes vs. no	NA ^a^	0.5 (−0.3, 1.2)
Additional 1 h/day of LPA	−0.2 (−0.5, 0.1)	−0.1 (−0.5, 0.2)
Additional 10 min/day of MVPA	0.7 (0.4, 1.0)	0.4 (0.04, 0.7)

^a^ Not applicable. CI, confidence interval; LPA, light-intensity physical activity; MVPA, moderate- and vigorous-intensity physical activity; NA, not applicable; OR, odds ratio.

## Data Availability

The data that support the findings of this study are available from https://www.cdc.gov/nchs/nnyfs/index.htm (accessed on 20 September 2022).

## Supplementary Materials

The following supporting information can be downloaded at: https://www.mdpi.com/article/10.3390/children9101433/s1, Table S1: Estimated time spent in various types of physical activity in a representative sample of U.S. preschool-aged children.
