# Supplementary material for: Physical Activity among U.S. Preschool-Aged Children: Application of Machine Learning Physical Activity Classification to the 2012 National Health and Nutrition Examination Survey National Youth Fitness Survey
Source: Children (Basel). 2022 Sep 21;9(10):1433. doi: 10.3390/children9101433 (PMC9600221; doi:10.3390/children9101433)
Supplement: Supplementary file 1 [file children-09-01433-s001.zip › children-1899361-supplementary.pdf]

Supplementary Data

Supplementary Table S1. Estimated time spent in various types of physical activity in a representative sample of U.S. preschool-aged children

	Run	Walk	Other MVPA	LPA	MVPA[a]	Total PA[b]
	Weighted mean (95% CI), minutes/day					
Language spoken at home						
English only (n=203)	4 (3, 5)	13 (12, 14)	9 (8, 11)	364 (343, 385)	27 (24, 29)	391 (367, 414)
At least some non-English (n=98)	4 (3, 5)	16 (13, 20)	10 (9, 12)	350 (324, 377)	31 (27, 35)	381 (351, 411)
Ratio of family income to poverty						
<1.0 (below the poverty line; n=112)	4 (3, 5)	16 (13, 19)	10 (8, 12)	363 (338, 388)	29 (24, 34)	392 (363, 422)
1.0 to <3.0 (n=110)	4 (2, 5)	15 (13, 16)	9 (8, 11)	370 (346, 393)	27 (25, 29)	397 (373, 422)
≥3.0 (n=79)	4 (3, 6)	12 (10, 14)	10 (8, 12)	345 (322, 368)	26 (21, 30)	371 (346, 397)

[a] Sum of run, walk, other MVPA.
[b] Sum of MVPA and LPA.
CI, confidence interval; LPA, light-intensity physical activity; MVPA, moderate- and vigorous-intensity physical activity; PA, physical activity.
